# Global survey of miRNAs and tRNA-derived small RNAs from the human parasitic protist *Trichomonas vaginalis*

**DOI:** 10.1186/s13071-020-04570-9

**Published:** 2021-01-29

**Authors:** Zhen-Sheng Wang, Hong-Chang Zhou, Chun-Yan Wei, Zhi-Hua Wang, Xiao Hao, Lian-Hui Zhang, Jing-Zhong Li, Zeng-Lei Wang, Heng Wang

**Affiliations:** 1 Department of Microbiology and Parasitology, Institute of Basic Medical Sciences, Chinese Academy of Medical Sciences, School of Basic Medicine, Peking Union Medical College, #5 Dong Dan San Tiao, Beijing, 100005 People’s Republic of China; 2 NHC Key Laboratory of Systems Biology of Pathogens, Institute of Pathogen Biology, Chinese Academy of Medical Sciences and Peking Union Medical College, Beijing, People’s Republic of China; 3 School of Medicine, Huzhou University and Huzhou Central Hospital, Huzhou, 313000 Zhejiang province China; 4 Department of Immunology, Guilin Medical University, Guilin, 541000 Guangxi province China; 5 Blood Chamber, Blood Station of Jinan, Jinan, 250000 Shandong province China; 6 NHC Key Laboratory of Echinococcosis Prevention and Control, #21 linkuo North Road, Chengguan District, Lhasa, 850000 Tibet Autonomous Region People’s Republic of China

**Keywords:** *Trichomonas vaginalis*, Transfer RNA, tRNA-derived small RNAs, tRFs, tRNA-halves, Trichomoniasis

## Abstract

**Background:**

Small non-coding RNAs play critical regulatory roles in post-transcription. However, their characteristics in *Trichomonas vaginalis*, the causative agent of human sexually transmitted trichomoniasis, still remain to be determined.

**Methods:**

Small RNA transcriptomes from *Trichomonas* trophozoites were deep sequenced using the Illumina NextSeq 500 system and comprehensively analyzed to identify *Trichomonas* microRNAs (miRNAs) and transfer RNA (tRNA)-derived small RNAs (tsRNAs). The tsRNA candidates were confirmed by stem-loop quantitative reverse transcription-PCR, and motifs to guide the cleavage of tsRNAs were predicted using the GLAM2 algorithm.

**Results:**

The miRNAs were found to be present in *T. vaginalis* but at an extremely low abundance (0.0046%). Three categories of endogenous *Trichomonas* tsRNAs were identified, namely 5′tritsRNAs, mid-tritsRNAs and 3′tritsRNAs, with the 5′tritsRNAs constituting the dominant category (67.63%) of tsRNAs. Interestingly, the cleavage site analysis verified both conventional classes of tRNA-derived fragments (tRFs) and tRNA-halves in tritsRNAs, indicating the expression of tRNA-halves in the non-stress condition. A total of 25 tritsRNAs were experimentally confirmed, accounting for 78.1% of all tested candidates. Three motifs were predicted to guide the production of tritsRNAs. The results prove the expression of tRFs and tRNA-halves in the *T. vaginalis* transcriptome.

**Conclusions:**

This is the first report of genome-wide investigation of small RNAs, particularly tsRNAs and miRNAs, from *Trichomonas* parasites. Our findings demonstrate the expression profile of tsRNAs in *T. vaginalis*, while miRNA was barely detected. These results may promote further research aimed at gaining a better understanding of the evolution of small non-coding RNA in *T. vaginalis* and their functions in the pathogenesis of trichomoniasis.
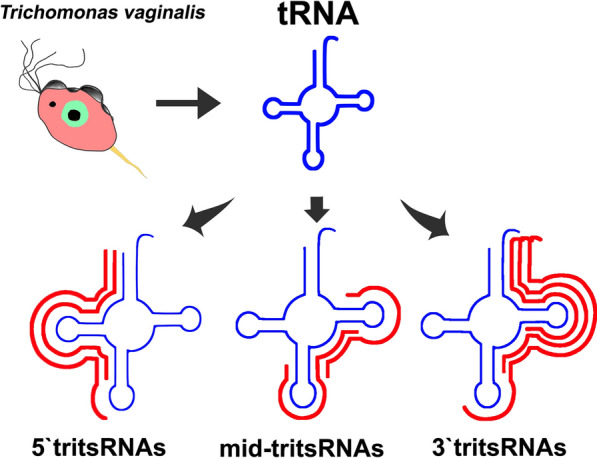

## Background

Small non-coding RNAs (snRNAs) have emerged as essential post-transcriptional regulators in a myriad of cellular and organismal processes and have consequently become an important focus of research in the past three decades. Since the discovery of microRNAs (miRNAs) in 1993 [1], the repertoire of snRNAs has greatly expanded through continuous characterization of new small RNAs from various origins, facilitated by the advance of deep sequencing technologies. In addition to such well-established snRNAs as miRNAs, endogenous small interference RNAs (endosiRNAs) and P-element induced wimpy testis (PIWI) protein-interacting RNAs (piRNAs), transfer RNA (tRNA)-derived snRNAs have remained a highly studied topic in recent years, and their important roles in a wide variety of biological and pathological situations have been increasingly recognized.

tRNA-derived small RNAs (tsRNAs) can be broadly categorized into two main classes that are likely dependent on their biogenesis: tRNA-halves and tRNA-derived fragments (tRFs). The conventional tRNA-halves are produced by a single ribonucleolytic cleavage within the anticodon loops of mature tRNAs under stress conditions, and thus they have also been referred to as tRNA-derived stress-induced small RNAs (tiRNAs) [2–6]. However, a recent study presented evidence of tRNA-halves being expressed in a non-stress condition [7], suggesting that the factors affecting the genesis of tRNA-halves might need to be re-evaluated. Endonucleolytic cleavages of both mature and precursor tRNAs near the D-,anticodon-,or TψC-arm lead to the generation of tRFs. Despite the need for a consistent nomenclature to describe the types of tRFs, they are typically classified into four subclasses: 5′tRFs, 3′tRFs (or 3′CCA tRFs), 3′UtRFs (tRF-1) and internal tRFs (itRFs or mid-tRFs) in terms of their positions mapped to tRNAs [8–10]. tRFs are constitutively and conservatively expressed in organisms from lower archaea to mammals; however, their biological roles and mechanisms of action remain largely unclear.

Research to date has associated tsRNAs with diverse human diseases and infections, revealing functions in the regulation of cell apoptosis, cell viability, RNA degradation, RNA stability, translational repression and cellular proliferation [6, 11–14]. Most interestingly, tRFs have the ability to behave like canonical miRNAs and regulate target gene expression through essential interactions with the Argonaute (AGO) family [15, 16], PIWI [17] or DICER proteins [18]. The similarity of tRFs and miRNAs has also resulted in misannotation and cross-mapping of these two groups. In some species that lack miRNA molecules, such as *Plasmodium* parasites, tRFs might act as alternative regulators to accomplish the intense post-transcriptional regulation needed during the rapid morphological change undergone by these organisms [10]. In the flagellated protozoan *Giardia lamblia*, the abundant tRFs detected were involved in the differentiation process, while miRNAs that had been previously reported were found to be absent or misannotated [19]. tRFs have also been discovered in other protozoan parasites, including *Tetrahymena* and *Trypanosoma cruzi* [20], as well as from exosomes of *Leishmania donovani* [21], implying their conservative expression in primitive eukaryotes, although their functions need to be further investigated.

*Trichomonas vaginalis* is an extracellular, unicellular flagellated protozoan parasite that belongs to the same class of Zoomastigophorea as *G. lamblia*. Infection of *T. vaginalis* leads to the occurrence of trichomoniasis, which remains the most prevalent non-viral sexually transmitted disease in humans, affecting 142.6 million people annually [22]. *Trichomonas vaginalis* parasites undergo dramatic biological changes even within the single trophozoite stage, including morphological change, DNA replication, multiple nucleus divisions, transposon activities and lateral gene transfer during differentiation. It is possible that the parasites take the advantage of certain snRNAs, such as tRNA-halves, tRFs or miRNAs, in these complicated changes. To date, miRNAs have only been reported by *in silico* prediction or hairpin-loop searching in *T. vaginalis* [23–25]. However, the misinterpretation of miRNAs discovered using the same methods in a closely related parasite, *G. lamblia* [19], hint at an ambiguous fate of *Trichomonas* miRNA molecules. Albeit a recent study revealed the occurrence of nine tRNA-halves in extracellular vesicles from *T. vaginalis* [7], their global expression profile in this parasite still remains to be revealed. It is therefore necessary to revisit the small RNAs in *T. vaginalis*, in particular tsRNAs and miRNAs, using advanced high-throughput deep sequencing to achieve a better understanding of the snRNAs in this parasite.

In the study reported here, we deeply sequenced the small RNA transcriptome from *Trichomonas* trophozoites. Based on our results from a genome-wide comprehensive analysis and experimental verification, we report for the first time the global identification and characterization of endogenous *Trichomonas* tsRNAs that may play a pivotal role in parasite development. In contrast, analysis of our sequencing data barely detected miRNAs.

## Methods

### *T. vaginalis* maintenance and RNA isolation

Three original clones (Tv01, Tv02 and Tv03) of *T. vaginalis* strains P7, which was isolated and cloned in 2012 and stored in liquid nitrogen until use, were maintained in Diamond’s media supplemented with 10% heat-inactivated bovine serum, penicillin (100 units/ml) and streptomycin (0.1 mg/ml) at 37°C without extra iron, as previously described [26]. The morphology of the parasites was checked daily to ensure the maintenance of typical pear-shaped trophozoites during culture. Parasite viability and density were monitored by Trypan blue exclusion on the hemocytometer. Fewer than 1 × 10^6^ trophozoites/ml was maintained during culture to avoid overgrowth. The pellet of each isolate was collected at the exponential phase of the trophozoite by centrifugation. Total RNA was extracted employing TRIzol Reagent (Invitrogen, Shanghai, China) and treated with DNase I to remove any genomic DNA contamination. RNA was then separated by urea-denatured 15% polyacrylamide gel electrophoresis, and bands of small RNAs with a length of 12–40 nucleotides (nt) were extracted and purified for further use.

### Small RNA library construction and deep sequencing

To avoid the interference of numerous post-transcriptional modifications in tRNA during complementary DNA (cDNA) synthesis and adapter ligation in RNA-sequencing, the following treatments were completed before library construction: (i) both 3′-aminoacyl deacylation and 2′, 3′-cyclic phosphate removal to 3′-OH for 3′ adaptor ligation; (ii) 5′-hydroxyl group phosphorylation to 5′-phosphorylation for 5′-adaptor ligation; (iii) m1A and m3C demethylation for efficient reverse transcription. The construction and subsequent sequencing of small RNA libraries were accomplished following commercial protocols. Briefly, small RNA molecules were ligated to 5′ and 3′ adaptors consecutively and then converted to cDNA by reverse transcription followed by PCR amplifications (RT-PCR). Approximately 2.34 fmol of RT-PCR products per sample were sequenced directly by the Illumina NextSeq 500 system (Illumina Inc. San Diego, CA, USA) at 50 bp single-read by Aksomics Inc. (Shanghai, China).

### Small RNA analysis and tsRNA identification

The quality of raw sequencing data was evaluated using FastQC (v0.11.7). The 3′ and 5′ adaptors were trimmed and reads shorter than 18 nt or longer than 40 nt were filtered out by Cutadapt (v1.17) and Python2 (v2.7.5) to remove any contamination and yield clean data. The clean dataset was further mapped to the genome of *T. vaginalis* G3 strain (TrichDB, release 47; http://trichdb.org/trichdb/) using BLASTN. The clean-read counts were normalized as a relative number per one million reads (RPM) and analyzed in both the total and unique read categories to indicate the abundance and diversity of reads, respectively. To investigate tRNA-derived segments, the data were further aligned to a total of 165 *T. vaginalis* tRNA genes downloaded from the TrichDB database (http://trichdb.org/trichdb/) by performing BLASTN alignment. The genome-wide expression intensity of the *T. vaginalis* tsRNAs was sequentially calculated with the “Build” function of Bowtie2 (v2.1.0.0) and the “coverageBed” function of Bedtools (v2.29.2). Data extraction of each type of tsRNAs was performed by compiled Perl (v5.22) codes. The statistical analysis for the Pearson correlation coefficients was carried out utilizing the “cor” function in R package stats. The ggplot2 package (v3.3.1) in R language (v3.5.0) was employed to create all the plots.

### Motif analysis

The motifs at the cleavage sites of tritsRNAs were predicted by using the GLAM2 algorithm in Gapped Local Alignment of Motifs (GLAM2 v1056) [22] with default parameters adopted, except that only the given strand was aligned.

### Stem-loop RT-PCR

The templates of total RNAs were reverse-transcribed into cDNAs utilizing the Goldenstar™ RT6 cDNA Synthesis Kit (Beijing TsingKe Biotech Co. Ltd., Beijing, China) following the manufacturer’s instructions. Specific stem-loop reverse transcription primers (Additional file 1: Table S1) were designed in accordance with the sequence of each tritsRNA candidate, as described previously [27, 28]. The PCR was carried out with an initial denaturation at 98 °C, 2 min; followed by denaturation at 98 °C/10 s, annealing at 55 °C/10 s and extension at 72 °C/15 s, for 35 cycles; with a final extension at 72 °C for 5 min. The PCR products were evaluated in a 12% polyacrylamide gel. The expected sizes of the PCR products were estimated by the length of each tsRNAs, with additional ~ 40 bp of nucleotides technically introduced into the stem-loop primers. Negative controls lacking DNase I-treated RNA or reverse transcriptase in the RT reactions or template in the PCR analyses were applied to validate the accuracy and specificity of the stem-loop RT-PCR analyses. The PCR primers are listed in Additional file 1: Table S2.

## Results

### Absence of miRNAs in *T. vaginalis*

**Fig. 1.**
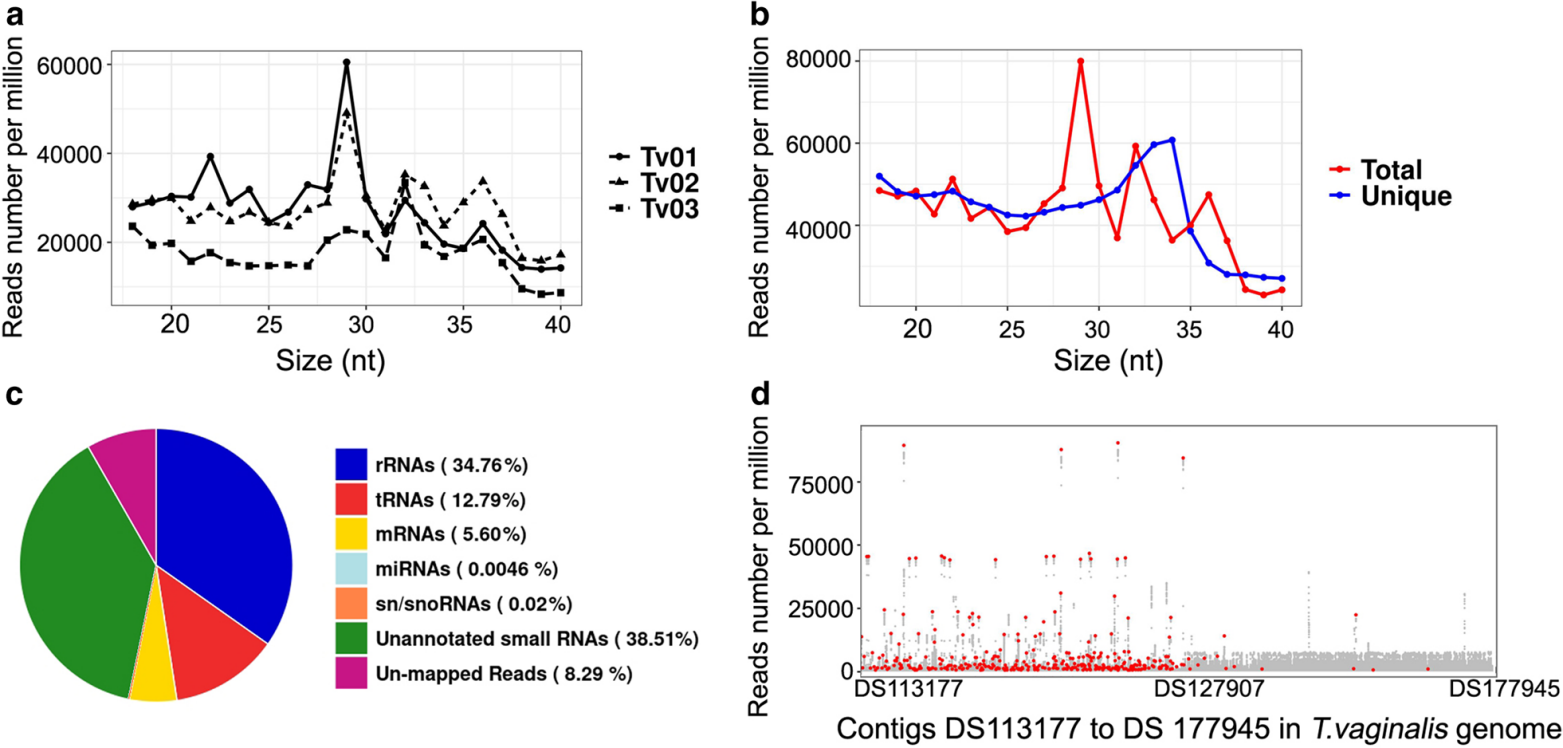
General features of 18- to 40-nt-long small RNAs from *Trichomonas vaginalis*. **a**,** b** Size distributions of small RNAs from individual samples (**a**) and pooled data (**b**). **c** Abundance of small RNA populations with reference to the *T. vaginalis* genomic annotation. **d** Genome-wide density analysis of small RNAs along all contigs of *T. vaginalis* genome. Red dots represent tsRNAs.* tsRNAs* Transfer RNA-derived small RNAs

Three *T. vaginalis* strains were employed for deep sequencing of the 18- to 40-nt small RNAs. As shown in Table 1, the three libraries yielded 6,303,530, 7,071,840 and 5,809,794 reads, respectively, that perfectly mapped to the *T. vaginalis* genome, accounting for an average of 91.76% of the clean data. The distribution of mapped reads from these three isolates was relatively consistent, all displaying three main peaks at 29, 32 and 36 nt (Fig. 1a). Consequently, these data were pooled together for further analysis to enhance the identification of any novel type of small RNAs. Apart from these three major peaks, in total reads, a single peak at 34 nt showed in unique reads (Fig. 1b). This feature was incredibly different from those of model organisms, which generally have demonstrated a peak at ~ 22 nt dominated by miRNAs in both the total and unique reads [29–31] and suggests that the composition of small RNAs in *T. vaginalis* might be divergent.

**Table 1.** Mapping and classification of reads against the *Trichomonas vaginalis* genome

Reads	Clone Tv01	Clone Tv02	Clone Tv03	Pooled data
Clean reads	6,909,748	7,811,640	6,186,244	20,907,632
Un-mapped reads	606,218	739,800	375,450	1,721,468
Mapped reads	6,303,530	7,071,840	5,809,794	19,185,164
mRNAs	190,790	322,082	651,191	1,164,063
5′UTR	3765	5245	7938	16,948
CDS	186,694	316,213	642,807	1,145,714
3′UTR	1219	1648	2095	4962
rRNAs	2,772,196	2,639,586	1,807,762	7,219,544
tRNAs	955,479	1,076,990	624,938	2,657,407
miRNAs	39,596	57,388	44,879	141,863
sn/snoRNAs	1331	1521	1532	4384
Unannoted small RNAs	2,344,138	2,974,273	2,679,492	7,997,903

CDS, Coding sequence; m/mi/r/tRNA, messenger/micro/ribosomal/transfer RNA, respectively; sn/snoRNA, small nuclear/nucleolar RNA, respectively; UTR, untranslated region

The data were then annotated, and the abundance of each type of small RNA was assessed in terms of its expression level. As illustrated in Fig. 1c, these small RNAs originated from ribosomal RNAs (rRNAs), tRNAs, messenger RNAs (mRNAs), small nuclear RNAs, small nucleolar RNAs (snoRNAs), as well as unannotated small RNAs. Unexpectedly, there was an extremely low abundance of miRNAs in our pool (0.0046%). Among the 27 miRNAs identified previously [23–25], only nine were detected in our deep sequencing data, and these had particular low counts, with the exception of ‘tvm-005’ derived from tRNA (Additional file 1: Table S3). In contrast, tsRNAs predominated after the rRNA-derived reads and accounted for 12.79% of clean reads. These molecules prevailed in *T. vaginalis* genomic contig DS113177 to DS127907, from where tRNA genes were mainly coded (Fig. 1d).

### Profiles of tsRNAs from *T. vaginalis*

**Fig. 2.**
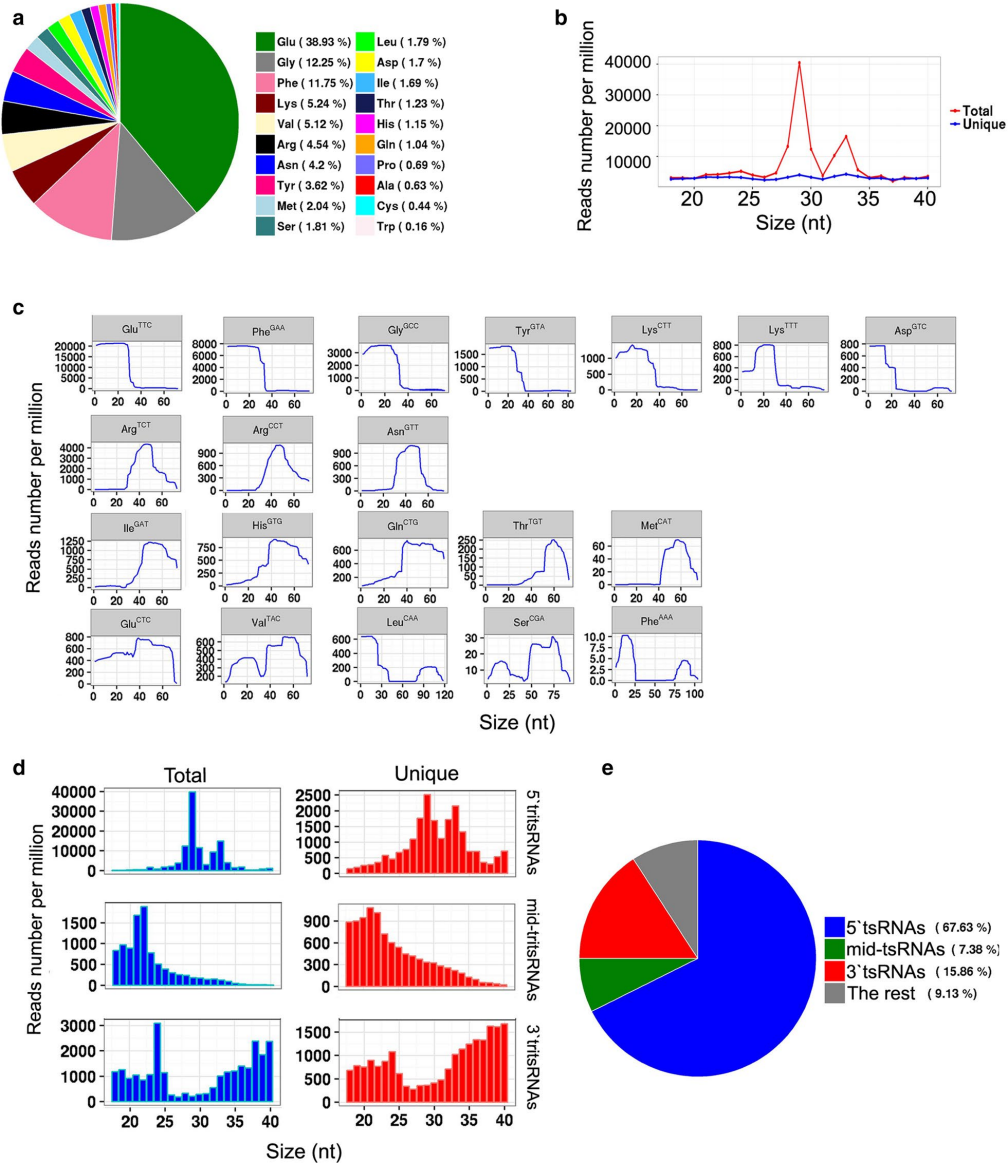
Profiles of tRNA-derived small RNAs from *T. vaginalis*. **a** Frequency of tsRNAs originating from tRNAs, **b** size distributions of tsRNAs, **c** coverage range analysis of tritsRNAs among the top 20 highest tsRNA-expressed tRNAs, **d** size distributions of three types of tritsRNAs in both the total and unique reads, **e** frequency of three categories of tsRNAs

To explore the expression profiles of tsRNAs, we analyzed their 20-amino acid parental tRNAs. Eight amino acids, namely Glu, Gly, Phe, Lys, Val, Arg, Asn and Tyr, were found to comprise up to 85% of all tsRNAs (Fig. 2a). This expression bias indicated that the tsRNAs were not generated by the random degradation of mature tRNAs. The distribution pattern of tsRNAs was investigated further and in addition to the distribution shown in Fig. 1b, there were only two conspicuous peaks, at ~ 29 and ~ 33 nt, that dominated in the total tsRNAs (Fig. 2b), implying multiple types of tsRNAs with diverse sizes in *T. vaginalis*. These tsRNA reads were therefore mapped to all 165 *Trichomonas* tRNAs. As expected, their biogenesis from the parental tRNAs was found to be relatively conserved, as previously reported [10, 19–21], with a large number of reads aligned to three main positions at the 5′ end, anticodon area and 3′ end of mature tRNAs (Fig. 2c). Consequently, these three types of *Trichomonas* tsRNAs were named 5′tritsRNAs, mid-tritsRNAs and 3′tritsRNAs, respectively. The homogeneity of these tritsRNAs based on their types and size distributions were investigated by plotting the RPM values against sizes in both the total and unique reads. Two peaks were observed for 5′tritsRNAs, at ~ 29 nt and ~ 33 nt; one peak was observed for mid-tritsRNAs, at ~ 21/22 nt; and two peaks were observed for 3′tritsRNAs, at ~ 24 nt and ~ 40 nt, respectively (Fig. 2d), displaying a great difference in their sizes. The double peaks that occurred in the plots of 5′tritsRNAs and 3′tritsRNAs suggested that there were at least two subgroups of tritsRNAs in each type.

We subsequently selected the top 20 tRNAs that produced high levels of tritsRNAs to further evaluate their expression divergence. Among these, seven primarily produced 5′tritsRNAs, three generated mid-tritsRNAs and five created 3′tritsRNAs; the remaining yielded tritsRNAs from multiple regions (Fig. 2c). 5′tritsRNAs were the dominant type (67.62%) of tritsRNAs, with mid-tritsRNAs and 3′tritsRNAs accounting for only 7.37% and 15.86%, respectively (Fig. 2e). No significant association between the abundance of any two types of tritsRNAs was found by Pearson correlation analysis (Additional file 2: Figure S1). The predominating tritsRNAs in each group were then examined by plotting the RPM values of all 60 tritsRNAs from these tRNAs against their sizes. A total of 32 tritsRNAs were identified to show consistent peaks, as shown in Fig. 2d, including nine 5′tritsRNAs, 12 mid-tritsRNAs and 11 3′tritsRNAs, as shown in Additional file 3: Figure S2, Additional file 4: Figure S3, Additional file 5: Figure S4 and in Table 2. These 32 tritsRNAs were then used for further classification and confirmation analysis.

**Table 2.** Classification of tritsRNA candidates

Category	Class	Parental tRNAs	Size (nt)
5'tritsRNAs	5′tritRFs	Glu^CTC^, Glu^TTC^, Lys^CTT^, Lys^TTT^, Val^GAC^, Tyr^GTA^	29
	5′tritRNA-halves	Phe^GAA^, Gly^GCC^, Val^TAC^	33
mid-tritsRNAs	mid-tritRFs	Arg^CCT^, Arg^TCT^, Asn^GTT^, Asp^GTC^, Gln^CTG^, His^GTG^, Ile^GAT^, Lys^CTT^, Met^CAT^, Val^GAC^, Phe^AAA^, Val^TAC^	21/22
3'tritsRNAs	3′tritRFs	Glu^TTC^, Val^TAC^, Lys^CTT^	24
	3′tritRFs	Arg^CCT^, Arg^TCT^, Glu^CTC^	33
	3′tritRNA-halves	Leu^CAA^, Val^GAC^, Thr^TGT^, Ile^GAT^, Met^CAT^	40

### Identification of tritRFs and tritRNA-halves

**Fig. 3.**
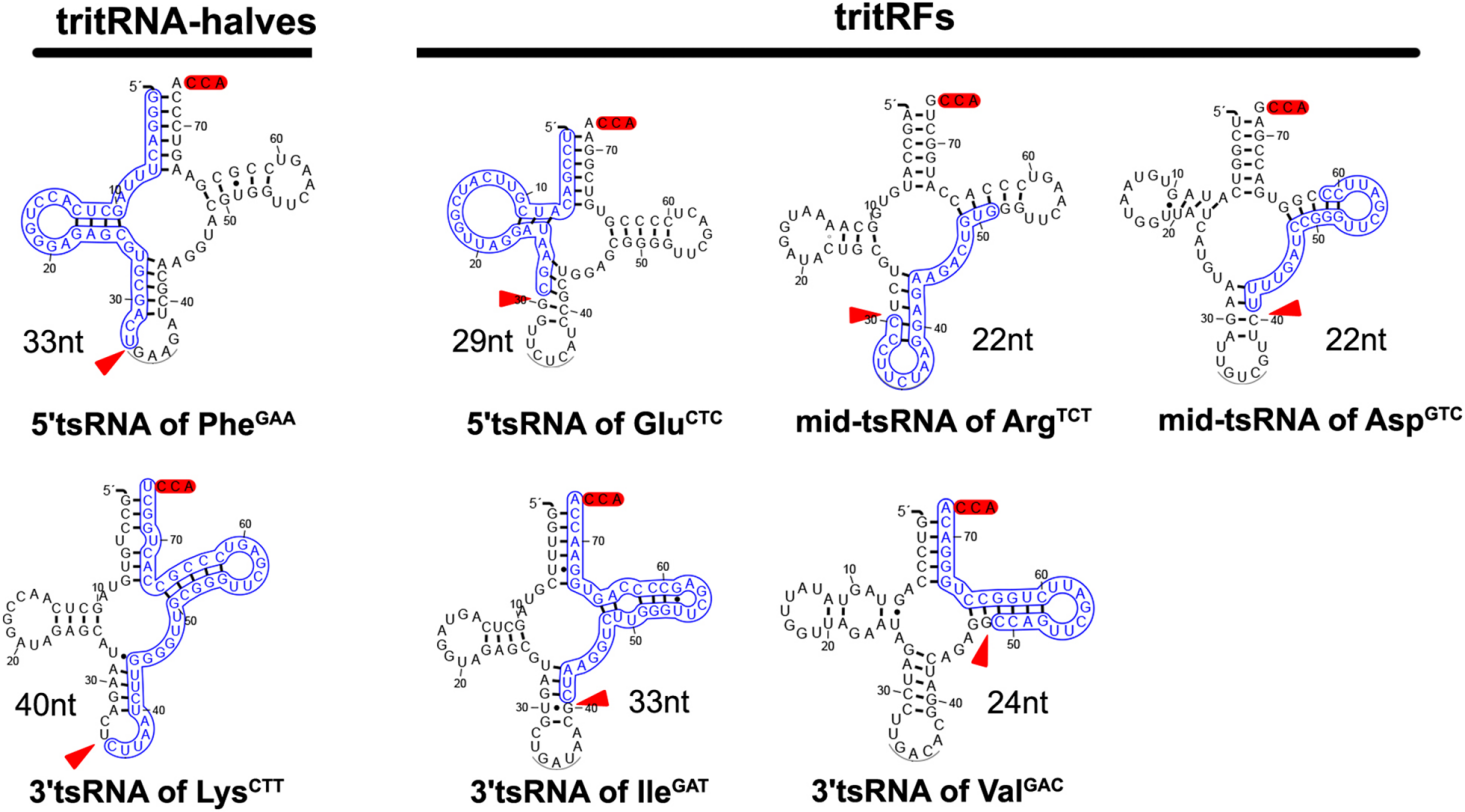
Cleavage sites of tritRNA-halves and of tritRFs in schematic mature tRNAs. The tritsRNA sequences are highlighted in blue, and cleavage sites are indicated by red triangles.* RF* RNA-derived fragments

tRFs, which are typically shorter than 32 nt [8], are the theoretical products of tsRNAs under ordinary conditions; however, our analysis identified longer products in both 3′ and 5′tritsRNAs. To further classify these tritsRNAs, we investigated their cleavage sites by aligning each of those 32 tritsRNAs to its parental tRNA. As illustrated in Fig. 3, two sites were identified that produced 5′tritsRNAs, with one occurring in the anticodon loop, generating the 33-nt 5′tritsRNAs, and one in the anticodon arm, yielding the 29-nt 5′tritsRNAs. The mid-tritsRNAs originated from two sets of combined endonucleolytic cleavages, both of which occurred in the arms neighboring the anticodon loop and TψC loop; however, one group covered the anticodon loop while the other involved the TψC loop. Three cleavage sites in anticodon loop, anticodon arm and TψC arm resulted in the generation of 3′tritsRNAs with sizes of 40, 33 and 24 nt, respectively. Therefore, 29-nt 5′tritsRNAs, mid-tritsRNAs and 24- and 33-nt 3′tritsRNAs were produced by combined cleavages other than that in the anticodon loop and belonged to the class of tritRFs, while two types (33-nt 5′tritsRNAs and 40-nt 3′tritRNAs) were generated by a single cleavage in the anticodon loop and thus were classified as tritRNA-halves (Table 2).

### Experimental confirmation of tritsRNA candidates and motif prediction

**Fig. 4.**
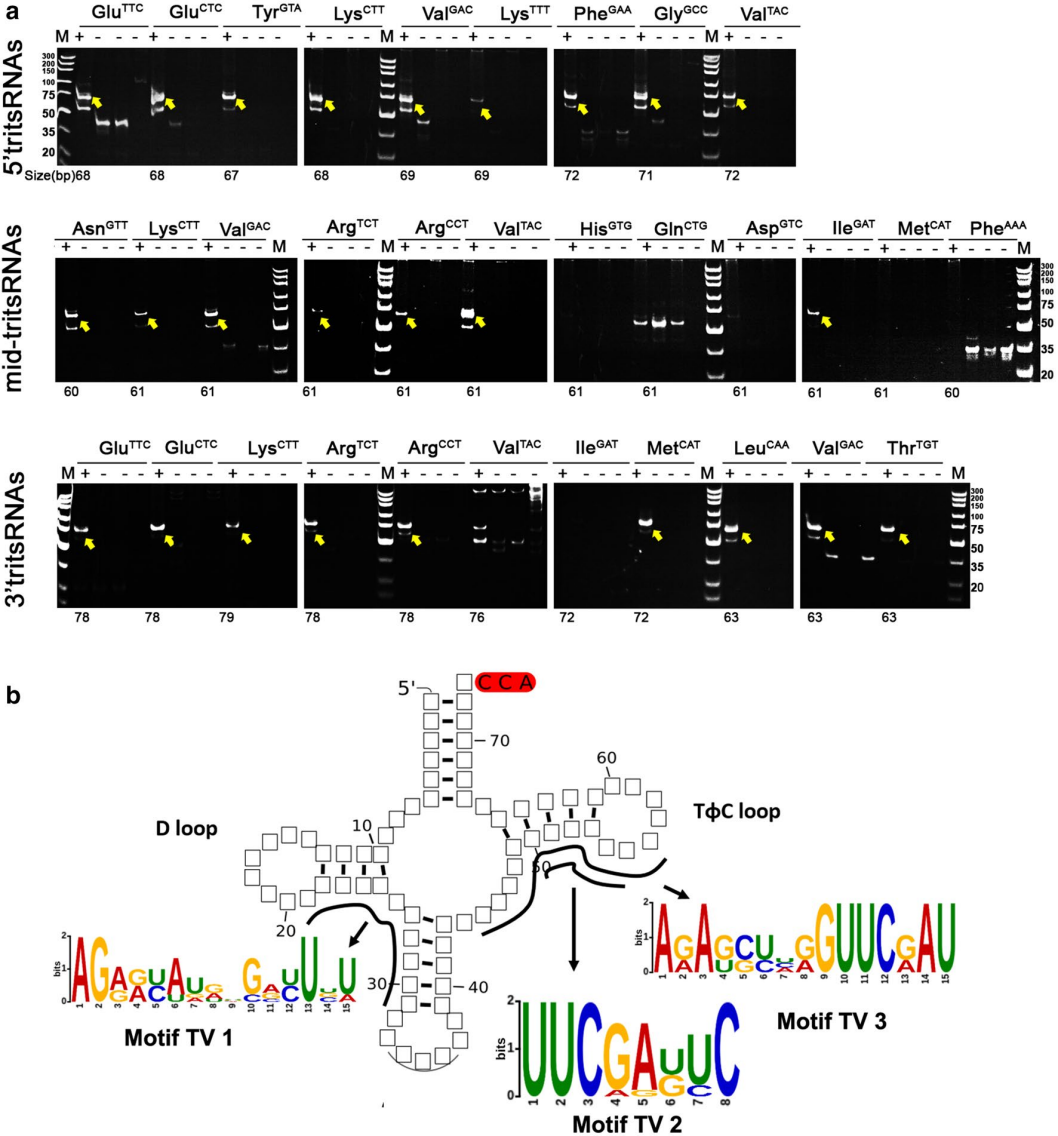
Experimental confirmation and motif prediction of tritsRNAs. **a** Reverse transcription (RT)-PCR validation of tritsRNA candidates. Each tritsRNA is shown in four adjacent lanes, which are from left to right: stem-loop RT-PCR with DNase-treated RNA template (left, “+”); without RNA template (left middle, “-”); without reverse transcriptase in RT reactions (right middle, “-”); and without template in the PCR reaction as negative control (right, “-”).* M* indicates the DNA ladder. The yellow arrows refer to the products with the expected size. **b** Schematic representation of the motif sequences and locations in the backbone of a mature tRNA.

We further experimentally evaluated the presence of tritRFs and tritRNA-halves in *T. vaginalis* to confirm our findings. Stem-loop RT-PCR successfully amplified 25 (9 5′tritsRNAs, 7 mid-tritsRNAs, 9 3′tritsRNAs) of these 32 candidates, corresponding to 78.1% of the candidates tested. This result proved that both tritRFs and tritRNA-halves were expressed in the *T. vaginalis* transcriptome (Fig. 4a).

The motifs around the cleavage sites that might guide the generation of tritsRNAs were then explored. Employing the GLAM2 algorithm, we successfully predicted that three motifs (Motifs TV1–3; Fig. 4b) would process tritsRNAs. Motif TV1 was located along the D-stem and anticodon-arm for the 3′ end cleavage of 5′tritsRNAs (29 nt). Motif TV2 and TV3 were both in the TψC stem to process the 3′ end cleavage of mid-tritsRNAs (22 nt) and 5′ end cleavage of 3'tritsRNAs (24 nt), respectively. Albeit both motif TV2 and TV3 contained the highly conservative ‘UUC’ triple-nucleotide element, they seemed to be controlling the generation of various types of tritsRNAs (Fig. 4b).

## Discussion

Despite the critical regulatory roles of snRNAs in post-transcription, our knowledge of their characteristics, biogenesis and functions in lower eukaryotes remains limited. The recent rapid development of high-throughput sequencing technology has greatly advanced research on small non-coding RNAs, revealing a variety of small RNAs, including those formerly misclassified tsRNAs. In this study, we used deep sequencing techniques and genome-wide studies to investigate small RNAs from the human parasite *T. vaginalis*. Strikingly, miRNAs occurred at a particularly low abundance (0.0046%) in our dataset. Although previous studies have reported 27 miRNAs based on *in silico* prediction or hairpin-loop searching, only nine were identified in our analysis. Two of them were derived from tRNAs and classified as 5′tritsRNA-Gln^TTG^ and 5′tritsRNA-Thr^TGT^, and one was apparently generated from 16S ribosomal RNA [23]. The extremely low abundance of miRNAs may indicate that their biological role is minimal or that they are not even present in *T. vaginalis*. It has been shown that miRNA is either absent or has no biological function in protists such as *Plasmodium falciparum* [10], *Giardia lamblia* [19] and *Leishmania* parasites [32], suggesting that these parasites likely employ other molecules to complete gene regulation. For example, tsRNAs have been reported to be involved in *Giardia lamblia* parasite differentiation [19]. In mammalian cells, tsRNAs have also been found to participate in translational regulation by binding a multienzyme complex [33], ribosomes [34] and stress granules [35] in an AGO-independent way. Since the AGO homolog has not been identified in *T. vaginalis*, it is thus possible that the tsRNA molecules in this parasite might adopt the AGO-independent manner and function in gene regulation.

In *T. vaginalis*, we identified three categories of tritsRNAs: 5′tritsRNAs, mid-tritsRNAs and 3′tritsRNAs. Of interest, both tRFs and tRNA-halves were classified from these tritsRNAs. tRNA-halves are generally believed to be the products of tRNAs under nutritional, biological or physicochemical stresses [2, 4, 36], while *T. vaginalis* parasites were cultured regularly at low density in our experiment. The presence of tRNA-halves was also noted in a recent study in which nine tRNA-halves were discovered from extracellular vesicles of the parasite *T. vaginalis* cultured under conditions of no external stress [7]. It is notable that no extra iron was included in both studies. Iron is essential in the development of *T. vaginalis*, while iron depletion having the potential to cause the accumulation of nitric oxide in a parasite’s cytoplasm, low expression of adhesins on the cell surface, formation of pseudocysts and prolonged survival [37–39]. Although the components in Diamond’s medium contain iron and could maintain the regular growth of parasites, the ideal level of iron needed remains unclear. Therefore, it is possible that variations in the level of iron in the culture medium place a nutritional stress on the parasite during development. To our knowledge, this is the first time that both tRFs and tRNA-halves have been reported in a protist organism under ordinary culture conditions, and the effect of iron in the generation of tsRNAs need to be further evaluated.

The mechanisms of tsRNAs biogenesis have yet to be revealed because the cleavage loci of known tRNA-specific nucleases still remain to be fully addressed. We therefore analyzed the motifs for tritsRNAs production. Since tRNA structure is highly conserved, there might be other factors involved in the genesis of tritsRNAs. In a tRNA molecule, the D- and T-loops interact with each other to support stabilization. It is thus possible that the motifs might be dependent on the spatial structure of tRNA and were composed of nucleotides that were not adjacent to each other along the sequence. Other unknown mechanisms might also take place in addition to motif recognition in tritsRNAs generation and need to be further elucidated.

## Conclusions

Taken together, this study has provided the first comprehensive evaluation of small non-coding RNAs in *T. vaginalis*. Previously reported miRNAs were not detected, indicating their absence or that they have little biological function in *T. vaginalis* parasites. Interestingly, three categories of tritsRNAs composed of both classes of tritRFs and tritRNA-halves were identified from *T. vaginalis* under a no stress condition. Future research is needed to understand whether these tsRNAs play a specific role in *T. vaginalis*. Our findings elucidate the profile of small RNAs and provide evidence of tRFs and tRNA-halves in *T. vaginalis*, which could lead to better understanding of the evolution of RNA processing in the early eukaryotes and the pathogenesis of trichomoniasis.

## Supplementary Information

**Additional file 1: Table S1.** Sequences of stem-loop reverse transcription primers. **Table S2.** Sequences of PCR primers. **Table S3.** Counts of reads mapped to known miRNA sequences.

**Additional file 2: Figure S1.** Scatter plots of tritsRNAs between each two categories.

**Additional file 3: Figure S2.** Size distribution of 5'tritsRNAs from the top 20 highest tsRNAs-expressed tRNA genes in total and unique reads.

**Additional file 4: Figure S3.** Size distribution of mid-tritsRNAs from the top 20 highest tsRNAs-expressed tRNA genes in total and unique reads.

**Additional file 5: Figure S4.** Size distribution of 3'tritsRNAs from the top 20 highest tsRNAs-expressed tRNA genes in total and unique reads.

## Data Availability

The datasets generated and analyzed during the current study are available in the GEO repository under the accession number GSE160464.

## Abbreviations

AGO: Argonaute protein
cDNA: Complementary DNA
mRNA: Messenger RNA
miRNA: MicroRNA
ncRNA: Non-coding RNA
piRNA: Protein-interacting RNA
PIWI: P-element induced wimpy testis
RPM: Relative number per one million reads
rRNA: Ribosomal RNA
RT-PCR: Quantitative reverse transcription-PCR
snRNA: Small non-coding RNA
snoRNA: Small nucleolar RNA
tRFs: Transfer RNA-derived fragments
tRNA: transfer RNA
tsRNAs: Transfer RNA-derived small RNAs
