# Supplementary material for: Global survey of miRNAs and tRNA-derived small RNAs from the human parasitic protist Trichomonas vaginalis
Source: Parasit Vectors. 2021 Jan 29;14:87. doi: 10.1186/s13071-020-04570-9 (PMC7844918; doi:10.1186/s13071-020-04570-9)
Supplement: Supplementary file 1 — Additional file 1: Table S1. Sequences of stem-loop reverse transcription primers. Table S2. Sequences of PCR primers. Table S3. Counts of reads mapped to known miRNA sequences. [file 13071_2020_4570_MOESM1_ESM.docx]

Table S1. The sequences of stem-loop reverse transcription primers.

| ID | RT primers |
| --- | --- |
| 501tsrnaRT | GTCGTATCCAGTGCAGGGTCCGAGGTTCGCACTGGATACGACACATATCC |
| 502tsrnaRT | GTCGTATCCAGTGCAGGGTCCGAGGTTCGCACTGGATACGACGCTTATCC |
| 503tsrnaRT | GTCGTATCCAGTGCAGGGTCCGAGGTTCGCACTGGATACGACCATGCTCTTC |
| 504tsrnaRT | GTCGTATCCAGTGCAGGGTCCGAGGTTCGCACTGGATACGACTATGCTCTATC |
| 505tsrnaRT | GTCGTATCCAGTGCAGGGTCCGAGGTTCGCACTGGATACGACGATCTATTC |
| 506tsrnaRT | GTCGTATCCAGTGCAGGGTCCGAGGTTCGCACTGGATACGACCAAAGCTC |
| 507tsrnaRT | GTCGTATCCAGTGCAGGGTCCGAGGTTCGCACTGGATACGACAGTCGCAC |
| 508tsrnaRT | GTCGTATCCAGTGCAGGGTCCGAGGTTCGCACTGGATACGACAAGGTGAAATTC |
| 509tsrnaRT | GTCGTATCCAGTGCAGGGTCCGAGGTTCGCACTGGATACGACAAGGATGTG |
| mid1tsrnaRT | GTCGTATCCAGTGCAGGGTCCGAGGTTCGCACTGGATACGACCGACTTCCAG |
| mid2tsrnaRT | GTCGTATCCAGTGCAGGGTCCGAGGTTCGCACTGGATACGACACCCGCAACC |
| mid3tsrnaRT | GTCGTATCCAGTGCAGGGTCCGAGGTTCGCACTGGATACGACCTGGCCTCTG |
| mid4tsrnaRT | GTCGTATCCAGTGCAGGGTCCGAGGTTCGCACTGGATACGACCACAGTCTTC |
| mid5tsrnaRT | GTCGTATCCAGTGCAGGGTCCGAGGTTCGCACTGGATACGACCACAGTCTTATC |
| mid6tsrnaRT | GTCGTATCCAGTGCAGGGTCCGAGGTTCGCACTGGATACGACTGGCCTTCC |
| mid7tsrnaRT | GTCGTATCCAGTGCAGGGTCCGAGGTTCGCACTGGATACGACCACGGATC |
| mid8tsrnaRT | GTCGTATCCAGTGCAGGGTCCGAGGTTCGCACTGGATACGACTGGATTTAG |
| mid9tsrnaRT | GTCGTATCCAGTGCAGGGTCCGAGGTTCGCACTGGATACGACGGGAATCG |
| mid10tsrnaRT | GTCGTATCCAGTGCAGGGTCCGAGGTTCGCACTGGATACGACGGGCTCGAAC |
| mid11tsrnaRT | GTCGTATCCAGTGCAGGGTCCGAGGTTCGCACTGGATACGACGGGCCCGAAC |
| mid12tsrnaRT | GTCGTATCCAGTGCAGGGTCCGAGGTTCGCACTGGATACGACCTTGGAATCG |
| 301tsrnaRT | GTCGTATCCAGTGCAGGGTCCGAGGTTCGCACTGGATACGACTTCCGATACG |
| 302tsrnaRT | GTCGTATCCAGTGCAGGGTCCGAGGTTCGCACTGGATACGACTTCCGACACG |
| 303tsrnaRT | GTCGTATCCAGTGCAGGGTCCGAGGTTCGCACTGGATACGACAGCCAGTGG |
| 304tsrnaRT | GTCGTATCCAGTGCAGGGTCCGAGGTTCGCACTGGATACGACCAGCCATGG |
| 305tsrnaRT | GTCGTATCCAGTGCAGGGTCCGAGGTTCGCACTGGATACGACAGCCCCAGG |
| 306tsrnaRT | GTCGTATCCAGTGCAGGGTCCGAGGTTCGCACTGGATACGACTGCCCCAAGC |
| 307tsrnaRT | GTCGTATCCAGTGCAGGGTCCGAGGTTCGCACTGGATACGACTGGTTCCAC |
| 308tsrnaRT | GTCGTATCCAGTGCAGGGTCCGAGGTTCGCACTGGATACGACTGGTTCCGCTG |
| 309tsrnaRT | GTCGTATCCAGTGCAGGGTCCGAGGTTCGCACTGGATACGACTAGGAGCTCTTG |
| 310tsrnaRT | GTCGTATCCAGTGCAGGGTCCGAGGTTCGCACTGGATACGACTGTCCCAGG |
| 311tsrnaRT | GTCGTATCCAGTGCAGGGTCCGAGGTTCGCACTGGATACGACTGCCTACGAC |

Table S2. The sequences of PCR primers.

| ID | Sequences of primers |
| --- | --- |
| 501tsrnaF | GACTTCCGATATCGTTCAGCG |
| 502tsrnaF | GATCTCCGACATCGTTCATC |
| 503tsrnaF | GATCCCGACCATAGCTCAG |
| 504tsrnaF | GATCGCCTGTGTAGCTCAAC |
| 505tsrnaF | GATCGTCCCAGTAGTATATTG |
| 506tsrnaF | GATCGCTTTCCTAGCTCAAC |
| 507tsrnaF | GATCGGGACTTTAGCTCAC |
| 508tsrnaF | GATCGCACCGCTGGTCTAATG |
| 509tsrnaF | GATCGCCCCAATAGTATATC |
| mid1tsrnaF | AGAGATCAGACTGTTAATCTG |
| mid2tsrnaF | GAGAGATCTCTTAATCTTGG |
| mid3tsrnaF | GAGAGATCCTTGACACGGATC |
| mid4tsrnaF | GAGAGATCCCCTTCTAAGGAG |
| mid5tsrnaF | GAGAGATCCCCTCCTAAGGAT |
| mid6tsrnaF | GAGAGATCCCTTTACACGGATG |
| mid7tsrnaF | GAGAGATCATTGTGGCTCAATA |
| mid8tsrnaF | GAGAGATCGACTCTGAATCC |
| mid9tsrnaF | GAGAGATCTTTTGATCCGGG |
| mid10tsrnaF | GAGAGATCCTAAGGTCTTGG |
| mid11tsrnaF | GAGAGATCCTAAGGTCTTGG |
| mid12tsrnaF | GAGAGATCTGATCCTGGTTCG |
| 301tsrnaF | GAGATCTTCATCCACAGGATC |
| 302tsrnaF | GAAGATCCTCATCCGCTGGAG |
| 303tsrnaF | GAAGATCCTTAATCTTGGGGTTG |
| 304tsrnaF | GAGATCCTAAGGAGAAGACTG |
| 305tsrnaF | GATCCTAAGGATAAGACTGTG |
| 306tsrnaF | AATCCACGGATGGAAGGCCAG |
| 307tsrnaF | CGATCCTAAGGTCTTGGGTTC |
| 308tsrnaF | CTGATCCTAAGGTCTTGGGTTC |
| 309tsrnaF | GAGAGATCTTGGTTCAATTCC |
| 310tsrnaF | GAGAGATCCCAGTTCGATTCTG |
| 311tsrnaF | GAGAGATCCCGGTTCAACTCC |
| general reverse | CCAGTGCAGGGTCCGAG |

Table S3. The counts of reads mapped to known miRNA sequences

| miRNA candidate IDs | Counts | | |
| --- | --- | --- | --- |
|  | TV01 | TV02 | TV03 |
| tvm-001 | 17 | 61 | 77 |
| tvm-002 | 84 | 104 | 90 |
| tvm-003 | 46 | 217 | 171 |
| tvm-004 | 5 | 11 | 9 |
| tvm-005 | 39425 | 56954 | 44513 |
| tvm-006 | 0 | 0 | 0 |
| tvm-007 | 0 | 0 | 0 |
| Tvm1_DS114515:7733-7712 | 0 | 2 | 5 |
| Tvm2_DS177933:152-132 | 0 | 0 | 0 |
| Tvm3_DS176142:693-673 | 0 | 0 | 0 |
| Tvm4_DS160029:395-416 | 0 | 0 | 0 |
| Tvm5_DS113666:32359-32338 | 0 | 0 | 0 |
| Tvm6_DS177803:248-269 | 7 | 11 | 9 |
| Tvm7_DS177310:460-481 | 1 | 0 | 1 |
| Tvm8_DS174663:730-750 | 0 | 0 | 0 |
| Tvm9_DS162040:804-784 | 0 | 0 | 0 |
| Tvm10_DS176757:229-209 | 0 | 0 | 0 |
| Tvm11_DS177474:213-232 | 0 | 0 | 0 |
| tva-miR-001 | 0 | 0 | 0 |
| tva-miR-002 | 0 | 0 | 0 |
| tva-miR-003 | 0 | 0 | 0 |
| tva-miR-004 | 0 | 0 | 0 |
| tva-miR-005 | 0 | 0 | 0 |
| tva-miR-006 | 11 | 28 | 4 |
| tva-miR-007 | 0 | 0 | 0 |
| tva-miR-008 | 0 | 0 | 0 |
| tva-miR-009 | 0 | 0 | 0 |
