# Supplementary figures and images for: Global survey of miRNAs and tRNA-derived small RNAs from the human parasitic protist Trichomonas vaginalis
Source: Parasit Vectors. 2021 Jan 29;14:87. doi: 10.1186/s13071-020-04570-9 (PMC7844918; doi:10.1186/s13071-020-04570-9)

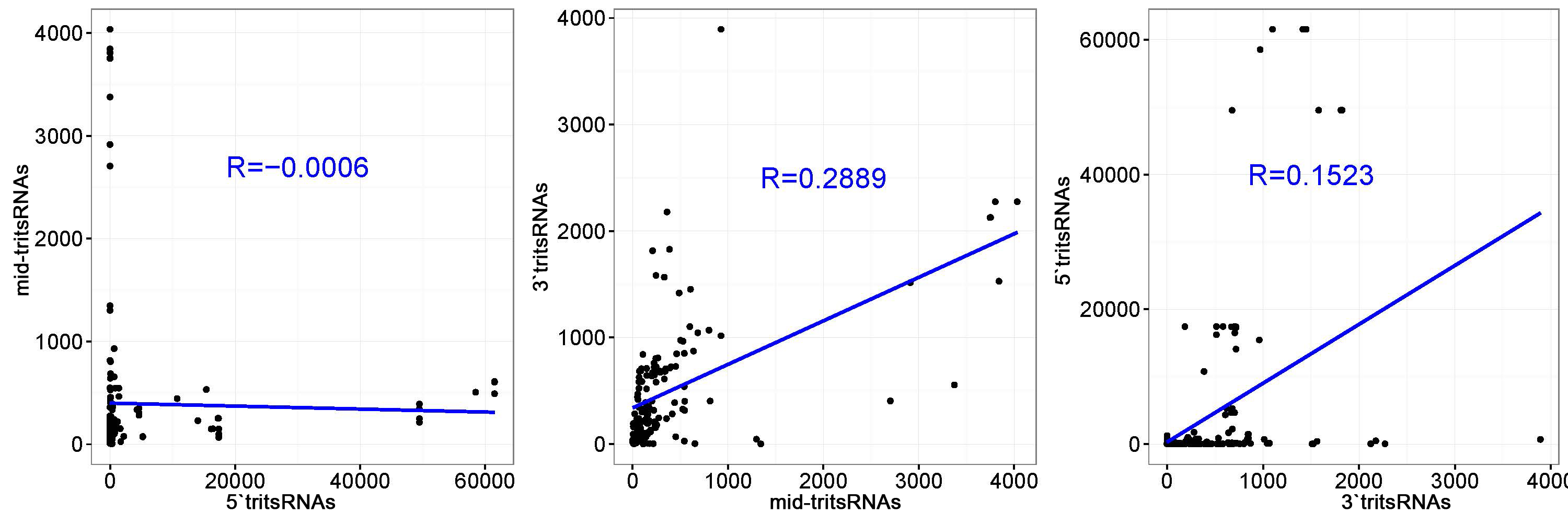

Supplement: Supplementary file 2 — Additional file 2: Figure S1. Scatter plots of tritsRNAs between each two categories. [file 13071_2020_4570_MOESM2_ESM.jpg]

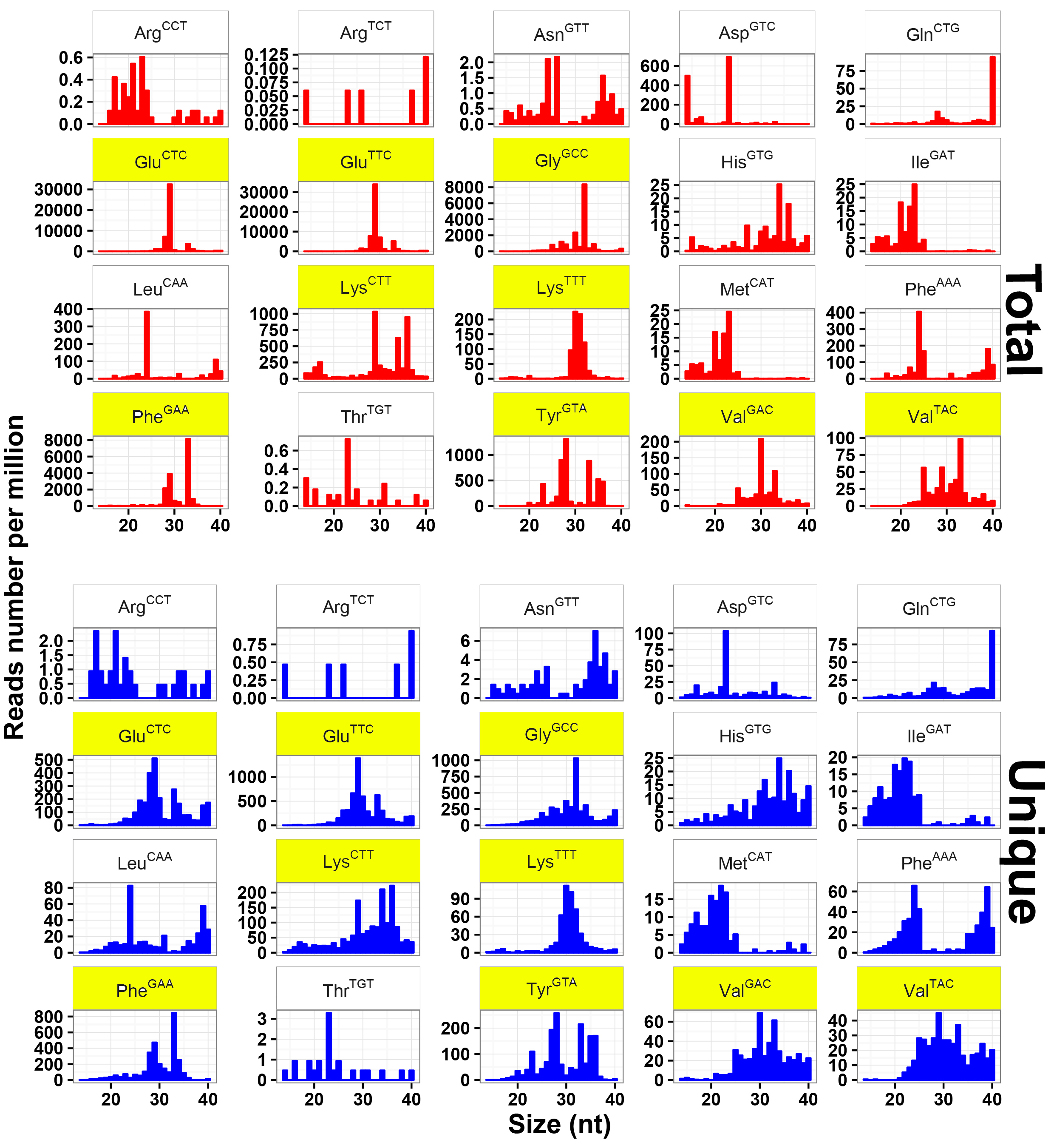

Supplement: Supplementary file 3 — Additional file 3: Figure S2. Size distribution of 5'tritsRNAs from the top 20 highest tsRNAs-expressed tRNA genes in total and unique reads. [file 13071_2020_4570_MOESM3_ESM.jpg]

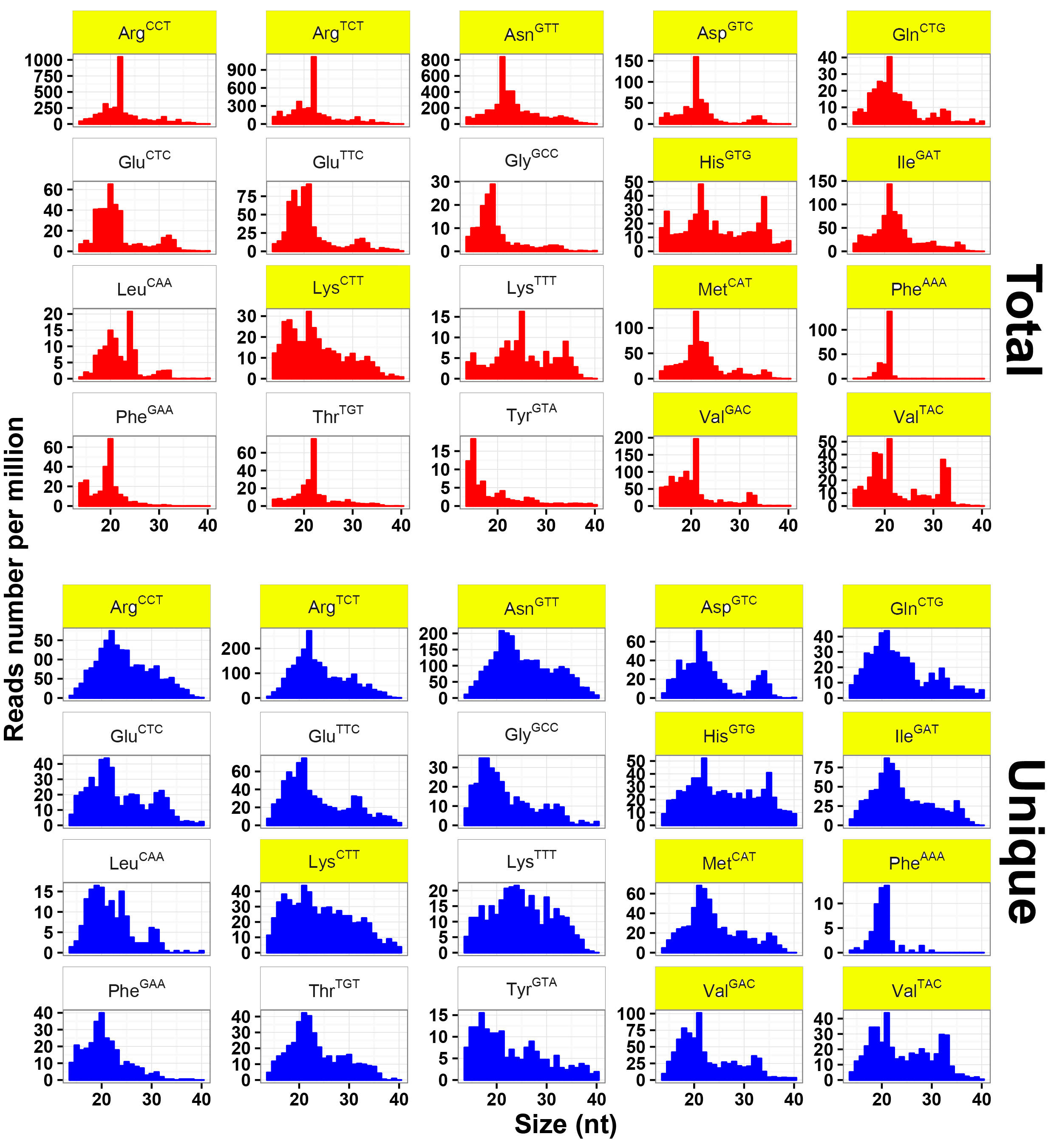

Supplement: Supplementary file 4 — Additional file 4: Figure S3. Size distribution of mid-tritsRNAs from the top 20 highest tsRNAs-expressed tRNA genes in total and unique reads. [file 13071_2020_4570_MOESM4_ESM.jpg]

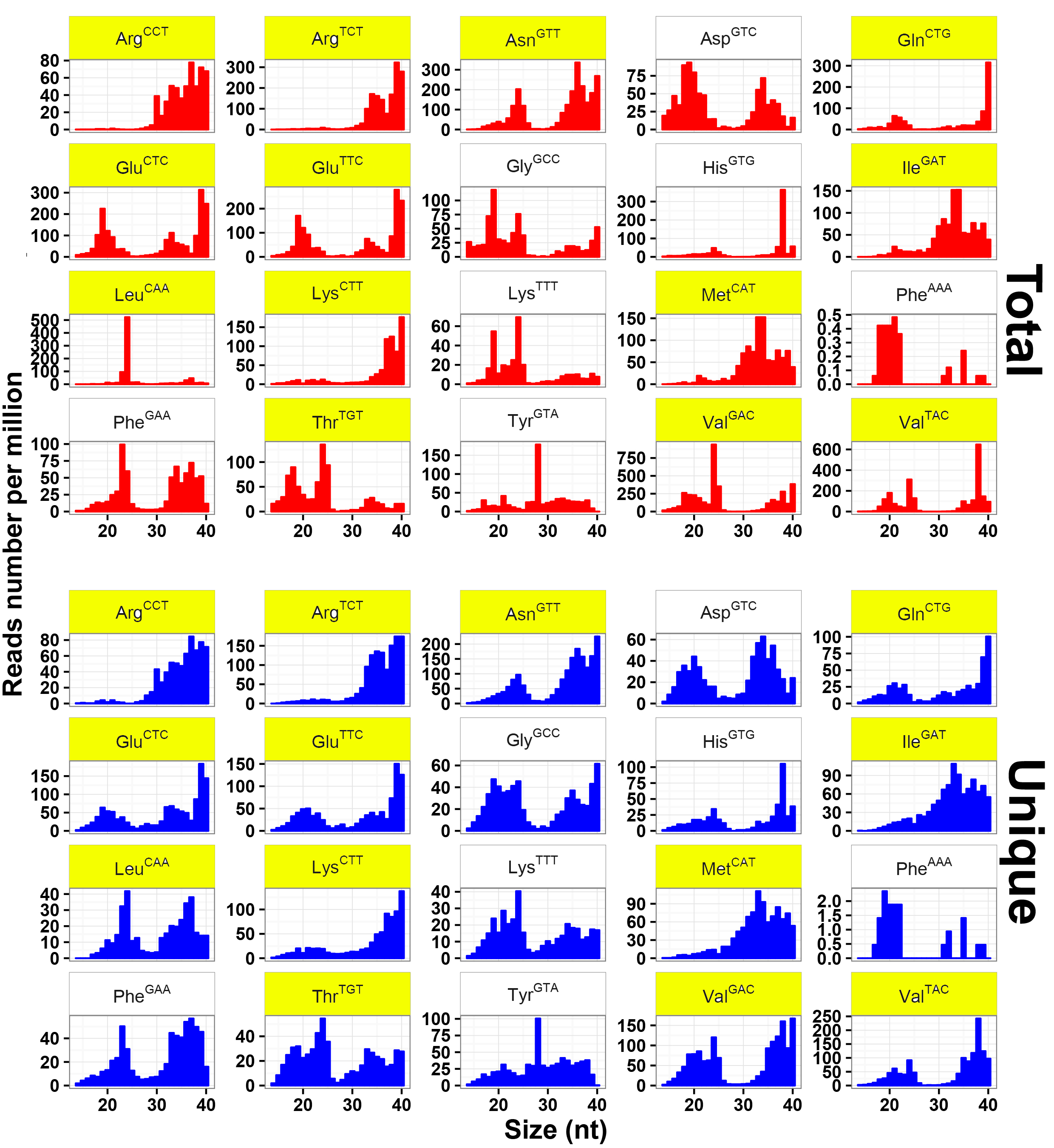

Supplement: Supplementary file 5 — Additional file 5: Figure S4. Size distribution of 3'tritsRNAs from the top 20 highest tsRNAs-expressed tRNA genes in total and unique reads. [file 13071_2020_4570_MOESM5_ESM.jpg]
